# Isoreticular Synthesis of Ionic Covalent Organic Frameworks for Enhanced SO_2_ Adsorption and Separation

**DOI:** 10.3390/molecules31091445

**Published:** 2026-04-27

**Authors:** Zhijie Liu, Shize Li, Jun Liang, Qiao Wu, Ruihu Wang

**Affiliations:** 1 Hebei Key Laboratory of Functional Polymer, School of Chemical Engineering and Technology, Hebei University of Technology, Tianjin 300401, China; 202321503051@stu.hebut.edu.cn (Z.L.); 202421503008@stu.hebut.edu.cn (S.L.); 2 State Key Laboratory of Structural Chemistry, Fujian Institute of Research on the Structure of Matter, Chinese Academy of Sciences, Fuzhou 350002, China

**Keywords:** ionic covalent organic frameworks, sulfur dioxide, carbon dioxide, adsorption

## Abstract

Achieving selective SO_2_ capture at low pressures is pivotal and challenging for possible flue gas desulfurization and air pollution control. In this study, we synthesized a series of ionic covalent organic frameworks (iCOFs) with *β*-ketoenamine linkages and sulfonic acid groups using a solvothermal method. TpPa-SO_3_H and TpBD-(SO_3_H)_2_ show a higher SO_2_ uptake of 4.46 and 5.24 mmol g^−1^ than TpPa-1 (4.24 mmol g^−1^) at 1 bar and 298 K, respectively, due to the combination of the good SO_2_ affinity of the polar sulfonic acid groups, higher pore volumes, and the good stability of *β*-ketoenamine COFs. TpBD-(SO_3_H)_2_ captured 2.83 mmol g^−1^ of SO_2_ at 0.1 bar and 298 K, which is 1.6 times higher than TpPa-1 (1.82 mmol g^−1^) under the same conditions. Notably, the IAST SO_2_/CO_2_ selectivity of TpBD-(SO_3_H)_2_ and TpPa-1 are 61 and 51, respectively, reflecting the impact of the incorporated SO_3_H groups’ higher affinity toward SO_2_. Notably, the multicomponent gas mixture breakthrough experiments confirm that TpBD-(SO_3_H)_2_ displays longer breakthrough time than TpPa-1 (987 vs. 311 min g^−1^). These *β*-ketoenamine iCOFs demonstrate nearly complete retention of crystallinity and porosity after exposure to dry or humid SO_2_. This work demonstrates that iCOFs are promising adsorbents for SO_2_ capture due to their high capacity, stability, and affinity for SO_2_ at low pressure.

## 1. Introduction

Sulfur dioxide (SO_2_) is a colorless, non-flammable gaseous species characterized by a pungent odor. Classified by the World Health Organization (WHO) as a highly toxic substance to human health, SO_2_ can be readily absorbed via respiratory inhalation and skin contact, and exposure to concentrations exceeding 100 ppm for merely a few minutes can be fatal [1,2]. SO_2_ released by the combustion of fossil fuels has caused serious impacts on human health and ecosystems [3,4,5]. It is challenging to selectively capture SO_2_ instead of CO_2_ with higher concentration (0.05–0.3% vs. 10–15 vol%) from flue gas [6]. To date, advanced flue gas desulfurization (FGD) technologies utilizing limestone scrubbing or amine scrubbing processes can remove approximately 90–95% of sulfur dioxide, but they are extremely energy-intensive [7]. Furthermore, the residual low-concentration SO_2_ (<500 ppm) may still contaminate downstream carbon dioxide scrubbing units [8,9,10] and irreversibly poison NO_x_ reduction catalysts [11] and CH_4_ combustion catalysts [12]. Thus, the development of new porous adsorbents for SO_2_ removal at low pressures is of great significance.

Given that SO_2_ possesses a larger kinetic diameter than CO_2_ (4.1 Å vs. 3.3 Å), a molecular sieving mechanism based on size exclusion is unattainable. Therefore, adsorption effect should be emphasized in the design of porous adsorbents for SO_2_ capture based on the different and superior physical properties of SO_2_ over CO_2_: dipole moment (1.63 vs. 0 D) and polarizability (47.7 × 10^−25^ vs. 26.5 × 10^−25^ cm^3^). Currently, activated carbon [13] adsorbents exhibit insufficient selectivity. Metal oxides [14] and zeolites [15] are difficult to be regenerated. Metal–organic frameworks (MOFs) demonstrate excellent adsorption capacity and selectivity; nevertheless, most MOFs suffer from poor stability [16,17,18,19,20]. Therefore, developing porous adsorbents with high SO_2_/CO_2_ selectivity and good stability presents a big challenge.

Covalent organic frameworks (COFs) represent a class of porous crystalline polymers featuring ultrahigh specific surface areas and highly designable structural units [21,22]. Their well-defined ordered architectures, unform open channels, and tunable skeletons render COFs as an ideal platform for elucidating structure–property relationships [23,24,25]. N-heterocyclic moieties [26,27,28], hydroxyl [29], and carboxylate groups [30] have been incorporated in COFs for SO_2_ adsorption and separation as well as SO_2_ sensing [31,32]. For example, Zhang et al. have reported an olefin-linked COF named NKCOF-12 featured with ultramicroporous structures and abundant nitrogen sites, thereby achieving high SO_2_ adsorption capacity and good desulfuration performance [32]. However, anionic COFs have not been explored for SO_2_ removal in this field. These iCOFs can be constructed from ionic monomers with sulfonic acid groups (–SO_3_H), which should be advantageous for preferential SO_2_ affinity and selective adsorption of SO_2_ due to the polarization capability of –SO_3_H groups. To the best of our knowledge, sulfonic acid functionalized COFs for the adsorption and separation of SO_2_ has not been reported.

**Figure 1:**
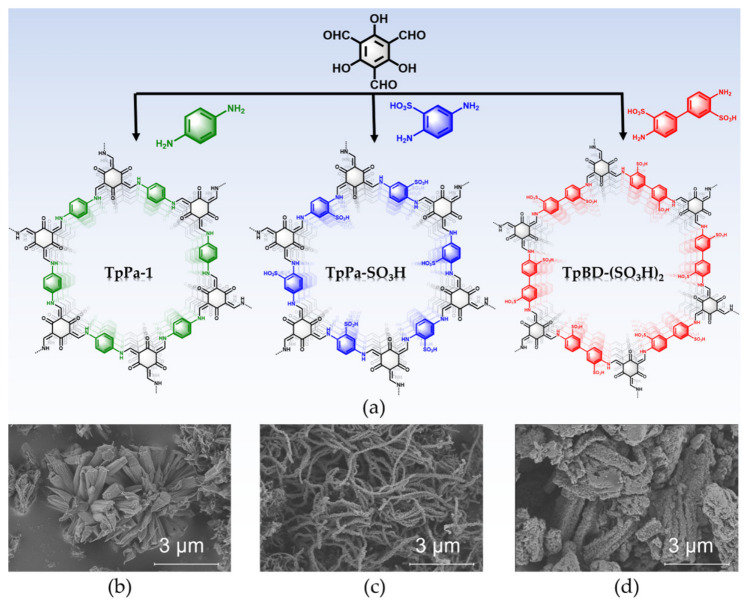
(**a**) Schematic representation of the synthesis of TpPa-1, TpPa-SO_3_H and TpBD-(SO_3_H)_2_ with hexagonal pores. SEM images of (**b**) TpPa-1, (**c**) TpPa-SO_3_H and (**d**) TpBD-(SO_3_H)_2_.

As a proof-of-concept study, herein, we report the isoreticular synthesis and structures of two sulfonic acid-functionalized COFs, namely TpPa-SO_3_H and TpBD-(SO_3_H)_2_ (Figure 1a), and their performance for the selective adsorption of SO_2_ over CO_2_, N_2_ and CH_4_. We introduced the –SO_3_H group into *β*-ketoenamine-based COFs via pre-synthetic strategy. This not only enhanced the interfacial polarity of the framework but also strengthened the interactions with SO_2_ molecules, thereby improving the affinity for SO_2_, especially under low-pressure conditions. Moreover, although the humid SO_2_ environmental conditions are of great significance in actual desulfurization scenarios, there are relatively few studies on the structural stability assessment in the literature regarding the use of COFs for SO_2_ removal. Our work demonstrated that these *β*-ketoenamine-based COFs exhibited good structural stability under both dry and humid SO_2_ conditions.

## 2. Results and Discussion

The synthetic routes of TpPa-1, TpPa-SO_3_H and TpBD-(SO_3_H)_2_ are depicted in Figure 1a, and all of them were prepared separately under similar solvothermal reaction conditions. Scanning electron microscopy (SEM) images show that TpPa-1 displays the flower-shaped morphology resulting from the aggregation of petals with lengths in the micrometer range (1–1.5 μm) (Figure 1b). TpPa-SO_3_H has nanofibrous morphology consisting of the interconnected tiny nanoparticles (Figure 1c). Moreover, TpBD-(SO_3_H)_2_ is composed of micrometer particles (Figure 1d).

**Figure 2:**
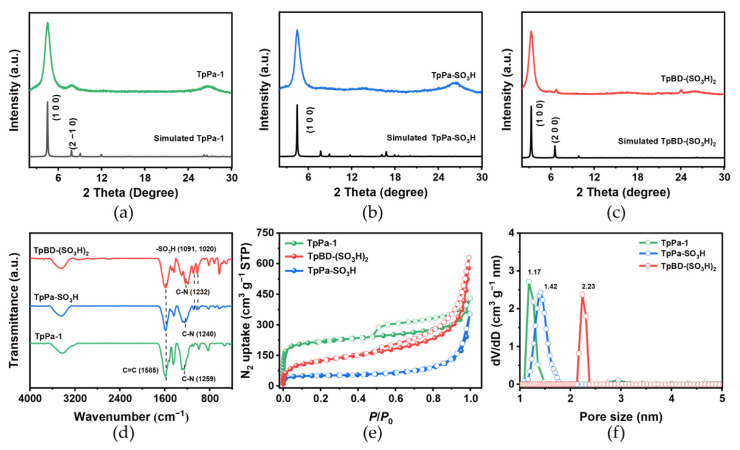
PXRD patterns of as-synthesized and simulated (**a**) TpPa-1, (**b**) TpPa-SO_3_H and (**c**) TpBD-(SO_3_H)_2_. (**d**) FT-IR spectra, (**e**) N_2_ adsorption-desorption isotherms with solid dots and white dots, respectively, and (**f**) pore size distributions based on nonlocal density functional theory (NLDFT) calculations for TpPa-1, TpPa-SO_3_H and TpBD-(SO_3_H)_2_.

The powder XRD pattern of TpPa-1 shows typical diffraction peaks at 2θ values of 4.7° and 8.1° (Figure 2a), which are assigned to the (100) and (2−10) planes from the A-A stacking model [33], while TpPa-SO_3_H exhibits intense peaks of (100) facet at 4.9° (Figure 2b) [34]. TpBD-(SO_3_H)_2_ exhibits intense peaks of (100) and (200) facets at 3.7° and 7.2° (Figure 2c) [35], respectively. These results confirm the good crystallinity of the obtained three COFs. The reference XRD patterns were taken from the literature [33,34,35].

The Fourier transform infrared (FT-IR) spectra of TpPa-SO_3_H and TpBD-(SO_3_H)_2_ are different from that of TpPa-1 (Figure 2d and Figures S1–S3). The C=C and C–N stretching bands occur at 1585 and 1232–1259 cm^−1^, respectively, which clearly corroborates the formation of the *β*-ketoenamine configuration in TpPa-1, TpPa-SO_3_H and TpBD-(SO_3_H)_2_. Furthermore, new bands at 1091 and 1020 cm^−1^ can be attributed to the stretching vibrations of -SO_3_H in TpPa-SO_3_H and TpBD-(SO_3_H)_2_.

To further probe the surface properties of these iCOFs with –SO_3_H groups, zeta potential measurements were carried out in pure water. TpPa-1 shows a slightly positive zeta potential of 5.5 mV, whereas TpPa-SO_3_H and TpBD-(SO_3_H)_2_ exhibit obviously negative zeta potentials of −31 mV and −36 mV, respectively (Figure S4). These results indicate that the presence of –SO_3_H groups markedly changes the surface charge properties of the frameworks and increases the surface polarity of the two iCOFs. Such polar surfaces should be advantageous to provide more adsorption sites and strengthen the interactions with polar SO_2_ molecules, especially under low-pressure conditions.

The permanent porosities were assessed by N_2_ sorption measurements at 77 K (Figure 2e). The N_2_ adsorption–desorption isotherms of all samples exhibited a combination pattern of type I and type IV [36]. The Brunauer–Emmett–Teller (BET) specific surface area of TpPa-1 is calculated to be 687 m^2^ g^−1^. With integrated –SO_3_H groups, the BET surface areas of TpPa-SO_3_H and TpBD-(SO_3_H)_2_ are determined to be 195 and 430 m^2^ g^−1^ (Table 1). The pore size distribution analyses show that the dominant pores are centered at 11.7, 14.2, 22.3 Å for TpPa-1, TpPa-SO_3_H and TpBD-(SO_3_H)_2_ (Figure 2f, Table 1), respectively. Thermogravimetric analysis (TGA) curves show that TpPa-1 is stable before 330 °C under N_2_ atmosphere (Figure S5). Compared to TpPa-1, TpPa-SO_3_H and TpBD-(SO_3_H)_2_ display a slightly higher thermal stability, remaining stable up to 400 °C.

The water contact angle measurements were performed to investigate the wettability of all samples in the form of pellets (Figure S6). The results indicate that all materials possess hydrophilic surfaces. It is noticed that after the incorporation of –SO_3_H groups in the two iCOFs, these frameworks become more hydrophilic than TpPa-1, probably due to the increased polarity of the two iCOFs’ surfaces, which is consistent with the results of zeta potential measurements (Figure S4). These polar iCOFs are expected to show selective adsorption performance toward SO_2_.

**Figure 3:**
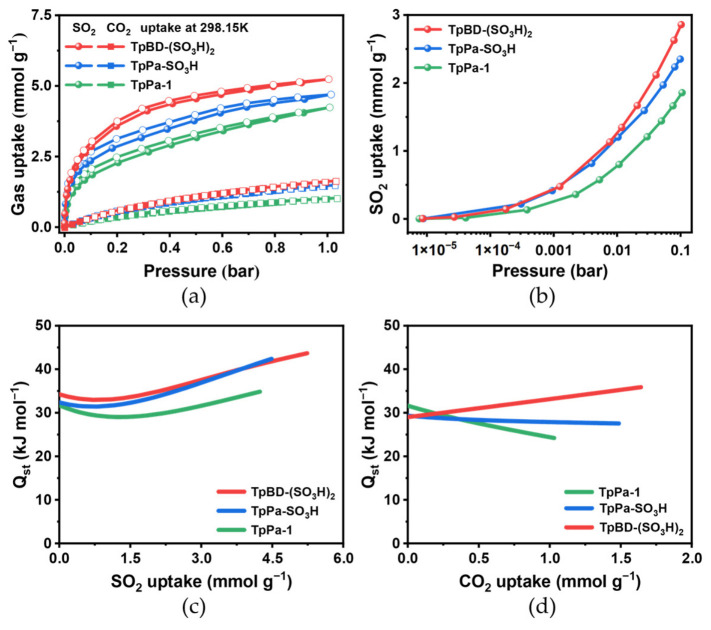
(**a**) SO_2_ and CO_2_ sorption isotherms of TpPa-1, TpPa-SO_3_H and TpBD-(SO_3_H)_2_ measured up to 1 bar at 298 K. (**b**) The enlarged SO_2_ adsorption at low pressure of 0–0.1 bar for better clarity of the onset of steep uptake. Isosteric enthalpy of adsorption of (**c**) SO_2_ and (**d**) CO_2_ on TpPa-1, TpPa-SO_3_H and TpBD-(SO_3_H)_2_ calculated by virial fitting of the single-component adsorption isotherms measured at 273 and 298 K, respectively (Figures S7–S10).

The SO_2_ adsorption isotherms of TpPa-1, TpPa-SO_3_H and TpBD-(SO_3_H)_2_ were collected at 298 K. At 1.0 bar, the SO_2_ adsorption capacities of TpPa-1, TpPa-SO_3_H and TpBD-(SO_3_H)_2_ were 4.24, 4.46, and 5.24 mmol g^−1^ (Figure 3a, Table 1). At 0.01 bar, TpPa-SO_3_H and TpBD-(SO_3_H)_2_ with –SO_3_H groups show significantly higher SO_2_ uptake capacities than TpPa-1 (1.19 mmol g^−1^ for TpPa-SO_3_H, 1.27 mmol g^−1^ for TpBD-(SO_3_H)_2_ and 0.78 mmol g^−1^ for TpPa-1) (Figure 3b, Table 1). Notably, the SO_2_ uptake of TpBD-(SO_3_H)_2_ at 298 K and 0.01 bar surpass the uptake in some well-known COFs, such as COF-701 [32] and TMT-TA [32]. As the pressure increased to 0.1 bar, the SO_2_ uptake of TpBD-(SO_3_H)_2_ rapidly rise to 2.83 mmol g^−1^, accounting for about 54% of the SO_2_ uptake. Similarly, TpPa-SO_3_H displays an adsorption capacity of 2.25 mmol g^−1^ at 0.1 bar, 50% of the total adsorption capacity. In contrast, TpPa-1 exhibits an adsorption capacity of 1.82 mmol g^−1^ at 0.1 bar, approximately 43% of the total adsorption capacity (Table 1). The observed relatively high SO_2_ uptake of TpBD-(SO_3_H)_2_ at low pressure (<0.1 bar) meets a prerequisite of potential adsorptive flue-gas desulfurization processes. The enhanced SO_2_ uptake of TpPa-SO_3_H and TpBD-(SO_3_H)_2_ in the low-pressure region should be reasonably associated with the introduction of polar –SO_3_H groups into the frameworks. This interpretation is further supported by the significantly negative zeta potentials of the sulfonated iCOFs (Figure S4). Compared with the non-sulfonated TpPa-1, the –SO_3_H-functionalized iCOFs provide more polar pore surfaces, which are favorable for strengthening the interactions with the polarizable SO_2_ molecules. Therefore, multiple adsorption sites (likely both carbonyl groups and sulfonate groups) might be responsible for the improved SO_2_ adsorption behavior under low pressures.

The SO_2_ adsorption isotherms at 273 K and 298 K were used to determine the isosteric enthalpy of SO_2_ adsorption (Δ*H*_ads_ = −Q_st_) by virial analysis (Figures S7 and S8) [37]. The −∆*H*_ads_ values near zero coverage (−Δ*H*_ads_^0^) in TpPa-1, TpPa-SO_3_H and TpBD-(SO_3_H)_2_ are 31.72, 32.36 and 34.26 kJ mol^−1^, respectively (Figure 3c). The −Δ*H*_ads_ values of the SO_3_H-functionalized COFs are slightly higher than that of TpPa-1, suggesting efficient host-guest interactions arising from the increased polarity of the two iCOFs.

The single-component CO_2_, CH_4_, and N_2_ adsorption isotherms for TpPa-1, TpPa-SO_3_H and TpBD-(SO_3_H)_2_ were measured at 298 K (Figures S9, S11 and S12). As expected, the two iCOFs exhibit slightly improved CO_2_ and CH_4_ adsorption performance than TpPa-1 up to 1 bar at 298K. Nevertheless, compared to TpPa-1, less N_2_ are adsorbed by TpPa-SO_3_H and TpBD-(SO_3_H)_2_ (Table S1). Notably, the SO_2_ adsorption curves of all COFs are much steeper than those of CO_2_ and CH_4_ probably due to the higher polarizability (47.7 × 10^−25^ cm^3^) and higher dipole moment (1.63 D) of SO_2_ [5]. It should be noted that the uptake amounts of CO_2_ and CH_4_ were much lower than the SO_2_ uptake on these COFs at 1.0 bar (Table S1). Through virial analysis of CO_2_ adsorption isotherms at 273 K and 298 K, the −Δ*H*_ads_^0^ of CO_2_ are determined to be 29.00, 29.32 and 31.61 kJ mol^−1^, respectively (Figure 3d and Figure S10). The generally higher −Δ*H*_ads_ values and steeper adsorption curves of SO_2_ than those of CO_2_ indicate the potential of these COFs for selective SO_2_ adsorption from gas mixtures. Overall, both the adsorption capacity of SO_2_ and isosteric heat of adsorption (−Δ*H*_ads_) by iCOFs are slightly increased than those of TpPa-1 under low-pressure conditions. These indicate that the introduction of the −SO_3_H groups helps to enhance the polarity of the framework and providing more electron-rich adsorption sites and higher pore volumes for SO_2_, thereby significantly improving the affinity and uptake amount of SO_2_ by these iCOFs.

**Figure 4:**
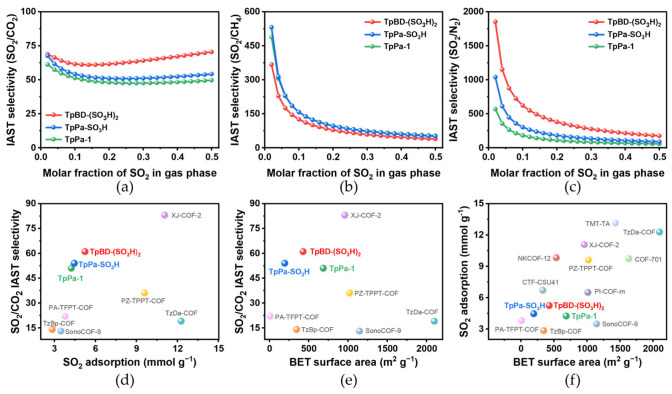
IAST selectivity of (**a**) SO_2_/CO_2_, (**b**) SO_2_/CH_4_ and (**c**) SO_2_/N_2_ for TpPa-1, TpPa-SO_3_H and TpBD-(SO_3_H)_2_ series as a function of SO_2_ molar fractions (0.02–0.5) at 1 bar and 298 K. Comparison of SO_2_ adsorption performance of TpPa-1, TpPa-SO_3_H, TpBD-(SO_3_H)_2_ and representative COFs. (**d**) Plot of SO_2_/CO_2_ selectivity against SO_2_ adsorption capacity at 1.0 bar. (**e**) Plot of SO_2_ adsorption capacity against BET surface area. (**f**) Plot of SO_2_/CO_2_ selectivity against BET surface area. Temperature for TpPa-1, TpPa-SO_3_H, TpBD-(SO_3_H)_2_ is at 298 K (Table S4).

To evaluate the selectivity of SO_2_ over CO_2_, CH_4_, and N_2_, ideal adsorbed solution theory (IAST) calculations were performed for binary gas mixtures as a function of variable SO_2_ molar fractions from 0.02 to 0.5 at 1 bar and 298 K. Considering the trace SO_2_ amount present in the flue gas, high SO_2_ selectivity over these gases is required for a realistic adsorptive gas desulfurization process. For a molar SO_2_/CO_2_ ratio of 10:90, the selectivity of TpPa-1 is 51, while TpPa-SO_3_H and TpBD-(SO_3_H)_2_ display an increased selectivity of 54 and 61 (Figure 4a and Table S3). To the best of our knowledge, the IAST SO_2_/CO_2_ selectivity value of TpBD-(SO_3_H)_2_ is relatively high among all the COFs materials reported so far (Figure 4d,f and Table S4). Meanwhile, TpBD-(SO_3_H)_2_ also possesses a high SO_2_/CH4 and SO_2_/N_2_ selectivity of 124 and 621, respectively, when the SO_2_/CH_4_ or SO_2_/N_2_ ratio is 10:90 (Figure 4b,c). Furthermore, at a CO_2_/N_2_ ratio of 10:90, the CO_2_/N_2_ selectivity of TpBD-(SO_3_H)_2_ and TpPa-SO_3_H are 70 and 355, respectively (Figure S13 and Table S3). Therefore, the experimental results have demonstrated the superiority of these iCOFs with SO_3_H groups over neutral TpPa-1 for enhanced selective SO_2_ capture in flue gas containing SO_2_, CO_2_ and N_2_. TpPa-SO_3_H and TpBD-(SO_3_H)_2_ also show enhanced selective CO_2_ capture performance than TpPa-1 (Figure S13).

For porous materials, it is reported that BET surface area and the pore volume are the main factors contributing to high SO_2_ adsorption capacity at high pressure. However, unlike high-pressure SO_2_ adsorption, the uptake at low pressure correlates with the affinity between SO_2_ and the adsorbent pore surface [18]. In our work, TpPa-1, TpPa-SO_3_H and TpBD-(SO_3_H)_2_ show moderate surface areas and an increased pore volume of 0.42, 0.51, and 0.96 cm^3^ g^−1^, which explains their moderate and enhanced SO_2_ uptake capacity at 1 bar and 298 K (Figure 4e). Moreover, the incorporation of SO_3_H in these iCOFs efficiently enhanced the SO_2_ uptake at low pressure range (<0.1 bar) by providing polar surfaces, leading to improved SO_2_/CO_2_ selectivities, which are even higher than some COFs with higher BET surface areas (Figure 4f).

To investigate the structural stability of TpPa-1, TpPa-SO_3_H and TpBD-(SO_3_H)_2_ towards SO_2_, all activated materials were exposed to dry SO_2_ and to humid SO_2_ for 6 h. The humid SO_2_ condition corresponds to a sealed air atmosphere containing 35 ppm SO_2_ and 75% RH (please see Section S5 and Figure S14 for details). The XRD patterns of all materials after dry and humid SO_2_ exposure remained, suggesting the retention of crystallinity without noticeable phase transformation (Figure S15). After all the materials were exposed to dry and humid SO_2_ environments, the FT-IR spectra showed very little change. This indicates that their structures were maintained and no obvious covalent bond breakage occurred (Figure S16). Interestingly, a new peak at 1350~1326 cm^−1^ in TpPa-1, TpPa-SO_3_H, and TpBD-(SO_3_H)_2_ after humid SO_2_ exposure is observed, which might be assigned to the asymmetric stretching vibrations of residual SO_2_ molecules [38,39]. The symmetric stretch of adsorbed SO_2_ (around 1144 cm^−1^) are not observed due to overlap with the strong vibration bands of TpBD-(SO_3_H)_2_ [40]. The BET surface areas of TpPa-1, TpPa-SO_3_H, and TpBD-(SO_3_H)_2_ after exposure to dry and humid SO_2_ remained 98%, 97% and 95% of the pristine COFs after dry and humid SO_2_ adsorption (Figures S17 and S18). To further evaluate the structural robustness, the materials were also exposed to humid SO_2_ for extended durations of 24 h and 72 h. No significant changes can be observed in the XRD patterns (Figure S15), FT-IR spectra (Figure S16), and BET surface areas (Figures S17 and S18). These results indicate that the frameworks can maintain their crystallinity, chemical integrity, and porosity under prolonged humid SO_2_ conditions, suggesting their potential for stable adsorption performance.

**Figure 5:**
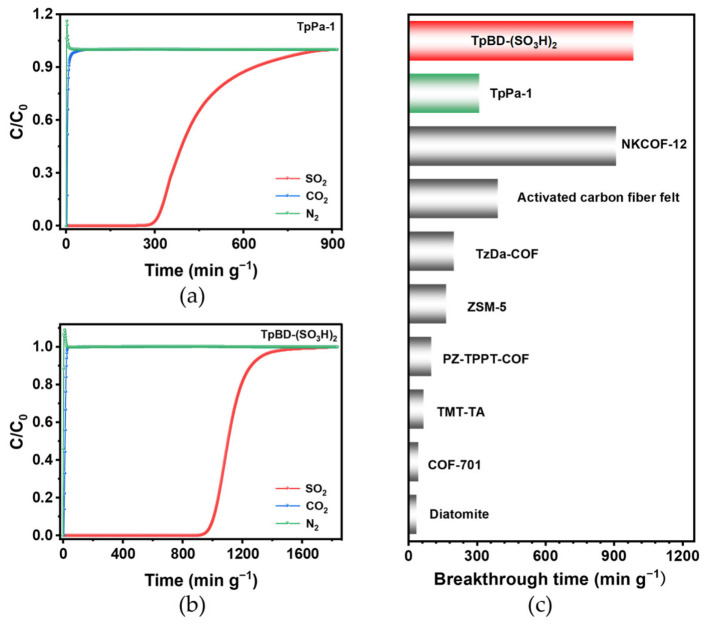
Experimental breakthrough curves of mixture gas SO_2_/CO_2_/N_2_ (2000 ppm + 14.8% + 85%) for (**a**) TpPa-1 and (**b**) TpBD-(SO_3_H)_2_. (**c**) The breakthrough time of TpPa-1, TpBD-(SO_3_H)_2_ and representative adsorbents.

Furthermore, the selective capture of SO_2_ from mixed gases is of great importance for practical applications. To further evaluate the separation performance under competitive conditions, dynamic breakthrough experiments were conducted. A typical gas mixture of SO_2_/CO_2_/N_2_ (2000 ppm of SO_2_ + 14.8% CO_2_ + 85% N_2_) was purged into a COF-packed column with an inlet flow rate of 8 mL min^−1^ at 298 K and 1 bar. As shown in Figure 5, N_2_ and CO_2_ broke through the column rapidly, while SO_2_ was retained in the adsorption bed for a significantly longer time, indicating the preferential adsorption of SO_2_. TpPa-1 exhibited an SO_2_ breakthrough time of ~311 min g^−1^ with a saturated breakthrough capacity of 0.31 mmol g^−1^ (Figure 5a). Notably, TpBD-(SO_3_H)_2_ displayed a much longer breakthrough time of 987 min g^−1^ and a higher saturated breakthrough capacity of 0.80 mmol g^−1^ (Figure 5b). Compared to the microporous neutral TpPa-1, the significantly enhanced breakthrough performance of TpBD-(SO_3_H)_2_ can be ascribed to the presence of –SO_3_H groups and higher pore volumes under competitive conditions. Moreover, TpBD-(SO_3_H)_2_ compares favorably with representative SO_2_ adsorbents reported in the literature (Figure 5c, Table S5). These results are consistent with the selectivity trend predicted from the IAST analysis.

## 3. Materials and Methods

### 3.1. Instrumentation

Powder X-ray diffraction (PXRD) patterns were collected on an X-ray diffractometer (Cu Kα radiation source, Miniflex600, Rigaku, Tokyo, Japan). Fourier transform infrared (FT-IR) spectra were recorded with KBr pellets using a Bruker TENSOR 27 (Bruker Corporation, Karlsruhe, Germany) spectrometer. The surface charge characteristics of the samples were characterized using the SURPASS 3 type Zeta potential analyzer (Anton Paar GmbH, Shanghai, China). Nitrogen adsorption and desorption isotherms were measured at 77 K using a surface area and porosity analyzer (JW-BK200, Jingwei Gaobo Instrument Co., Ltd., Beijing, China). The samples were degassed at 120 °C for 10 h before the measurements. Specific surface areas were calculated from the adsorption data using the Brunauer–Emmett–Teller (BET) equation. The pore size distributions were obtained using nonlocal density functional theory (NLDFT) calculations with an “N_2_-Tarazona, cylinder” model. Thermogravimetric analysis (TGA) was carried out on a TG209F1 Libra instrument (NETZSCH Group, Bayern, Germany) by heating the samples from 30 to 800 °C in a dynamic nitrogen atmosphere with a heating rate of 10 °C min^−1^. The water contact angle was observed using a water contact angle analyzer (DSA100, KRÜSS, Hamburg, Germany). Scanning electron microscopy (SEM) images were obtained on a MIRA LMS microscope (Tescan Group a.s., Bohunice, Czech Republic) operated at an accelerating voltage of 3.0 kV. The humid SO_2_ atmosphere was monitored using a portable SO_2_ detector (GT903-SO_2_-B, Kornuo Electronic Technology Co., Ltd., Shenzhen, China). 

Comparative SO_2_, CO_2_, CH_4_ and N_2_ gas adsorption experiments at 273 and 298 K:

Before each experiment, samples were activated for 12 h and at a minimum of 393 K under a vacuum < 5 × 10^−3^ mbar. SO_2_, and CH_4_ sorption experiments were measured at 273 and 298 K on an advanced corrosive gas adsorption and micropore analyzer (BSD-660MC, Beishide Instrument Technology Co., Ltd., Beijing, China) instrument within a pressure range of 1 × 10^−3^ to 1.0 bar. CO_2_, and N_2_ sorption experiments were measured at 273 and 298 K on an advanced measurement instrument (JW-BK330C, Jingwei Gaobo Instrument Co., Ltd., Beijing, China) within a pressure range of 1 × 10^−3^ to 1.0 bar. The breakthrough separation experiments were conducted in a multi-constituent adsorption breakthrough curve analyzer (BSD-MAB, Beishide Instrument Technology Co., Ltd., Beijing, China) under ambient conditions (298 K, 1 bar) using a gas mixture of 2000 ppm SO_2_ + 14.8% CO_2_ +85% N_2_. All used gases were of ultra-high purity (99.999%) and supplied by Sizhiqiti, Tianjin, China.

### 3.2. Chemicals

The 2,4,6-Triformylphloroglucinol (Tp) (98%), *p*-Phenylenediamine (Pa) (99%), 2,5-Diaminobenzenesulfonic acid (Pa-SO_3_H) (98%) and 4,4′-diamino-3,3′-biphenyl-disulfonic acid (BD-(SO_3_H)_2_) (97%) were obtained from the commercial supplier Adamas-beta (China), and were used without further purification. All solvents were purchased from commercial suppliers with a minimum purity of 99.8%.

### 3.3. Synthesis of TpPa-1

TpPa-1 was synthesized according to the modified literature procedures [33]. Tp (63.00 mg, 0.30 mmol), Pa (48.70 mg, 0.45 mmol), 1.5 mL of mesitylene and 1.5 mL of 1,4-dioxane were successively added to a Pyrex tube. This mixture was dispersed by ultrasonication for 10 min to get homogenous dispersion. Subsequently, 0.5 mL of CH_3_CO_2_H (3 mol L^−1^) was added to the tube. After the mixture was sonicated for 10 min, the tube was frozen under liquid N_2_ bath, and air was removed through three-pump-thaw cycles. Then, the tube was sealed off and put in an oven at 120 °C for 72 h. Reddish-brown powder was obtained by filtration after the mixture cooling down to room temperature. The powder was washed with tetrahydrofuran (THF), and further purified by Soxhlet extraction with THF over 24 h. The resultant reddish-brown solid was dried in a vacuum oven at 60 °C for 24 h.

### 3.4. Synthesis of TpPa-SO_3_H

TpPa-SO_3_H was synthesized according to the modified literature procedures [34]. Tp (63.00 mg, 0.30 mmol), Pa-SO_3_H (84.70 mg, 0.45 mmol), 2.7 mL of mesitylene and 0.3 mL of 1,4-dioxane were successively added to a Pyrex tube. This mixture was dispersed by ultrasonication for 10 min to get homogenous dispersion. Subsequently, 0.3 mL of CH_3_CO_2_H (6 mol L^−1^) was added to the tube. After the mixture was sonicated for 10 min, the tube was frozen under liquid N_2_ bath, and air was removed through three-pump-thaw cycles. Then, the tube was sealed off and put in an oven at 120 °C for 72 h. Reddish-brown powder was obtained by filtration after the mixture cooled down to room temperature. The powder was washed with acetone, and further purified by Soxhlet extraction with acetone over 24 h. The resultant reddish-brown solid was dried in a vacuum oven at 60 °C for 24 h.

### 3.5. Synthesis of TpBD-(SO_3_H)_2_

TpBD-(SO_3_H)_2_ was synthesized according to the modified literature procedures [35]. Tp (63.00 mg, 0.30 mmol), BD-(SO_3_H)_2_ (155.00 mg, 0.45 mmol), 2.4 mL of mesitylene and 0.6 mL of 1,4-dioxane were successively added to a Pyrex tube. This mixture was dispersed by ultrasonication for 10 min to get homogenous dispersion. Subsequently, 0.5 mL of CH_3_CO_2_H (6 mol L^−1^) was added to the tube. After the mixture was sonicated for 10 min, the tube was frozen under liquid N_2_ bath, and air was removed through three-pump-thaw cycles. Then, the tube was sealed off and put in an oven at 120 °C for 72 h. Reddish brown powder was obtained by filtration after the mixture cooling down to room temperature. The powder was washed with acetone, and further purified by Soxhlet extraction with acetone over 24 h. The resultant reddish-brown solid was dried in a vacuum oven at 60 °C for 24 h.

## 4. Conclusions

We have synthesized two *β*-ketoenamine-based ionic COFs with high thermal and chemical stability for the demanding selective SO_2_ capture at low pressures. Compared to the neutral COF without sulfonic acid group, it is found that the incorporation of sulfonic acid groups in these iCOFs could provide more polar surfaces for SO_2_ adsorption, and achieve higher SO_2_ affinity, higher SO_2_ uptake capacity (5.24 mmol g^−1^, 298 K, 1 bar) and improved SO_2_/CO_2_ selectivity (61, 298 K) in TpBD-(SO_3_H)_2_. The iCOFs also display enhanced SO_2_ capture performance than the neutral counterpart COF at low pressures of 0.001–0.01 bar. Additionally, all β-ketoenamine COFs exhibit excellent structural stability toward both dry and humid SO_2_. Considering the synthetic scalability, excellent stability and tunability of *β*-ketoenamine COFs [41], we believe that more ionic COFs can be developed for SO_2_ capture.

## Figures and Tables

**Table 1:** Porosity characteristics of TpPa-1, TpPa-SO_3_H and TpBD-(SO_3_H)_2_ and the results of SO_2_ adsorption at 298 K.

Material	BET-Surface Area ^1^ [m^2^ g^−1^]	Pore Width ^2^ [Å]	SO_2_ Uptake (298 K) [mmol g^−1^] at:			SO_2_/CO_2_ Selectivity ^3^ at SO_2_/CO_2_ Molar Ratio:	
			0.01 Bar	0.1 Bar	1.0 Bar	0.1	0.5
TpPa-1	687	1.00–1.59	0.78	1.82	4.24	51	49
TpPa-SO_3_H	195	1.12–1.81	1.19	2.25	4.46	54	54
TpBD-(SO_3_H)_2_	430	2.13–2.38	1.27	2.83	5.24	61	70

^1^ Obtained from five adsorption points in the pressure range 0.001 < P/P_0_ < 0.05. ^2^ Pore widths from pore size distribution are measured by N_2_ sorption at 77 K. ^3^ See Section S4 in the SI for the CO_2_ sorption data.

## Data Availability

The original contributions presented in this study are included in the article/Supplementary Materials. Further inquiries can be directed to the corresponding author.

## Supplementary Materials

The following supporting information can be downloaded at: https://www.mdpi.com/article/10.3390/molecules31091445/s1, Section S1: Isosteric enthalpy of adsorption; Section S2: Ideal adsorbed solution theory (IAST) Selectivity; Section S3: Characterization and analysis; Section S4: SO_2_, CO_2_, CH_4_ and N_2_ gas adsorption experiments; Section S5: Stability of crystallinity and porosity after dry and humid SO_2_ exposure; Section S6: Breakthrough performance; Section S7: References [26,27,28,29,30,31,32,37,42]; Figure S1: FT-IR spectra of Tp, Pa and TpPa-1; Figure S2: FT-IR spectra of Tp, Pa-SO_3_H and TpPa-SO_3_H; Figure S3: FT-IR spectra of Tp, BD-(SO_3_H)_2_ and TpBD-(SO_3_H)_2_; Figure S4: Zeta potential of TpPa-1, TpPa-SO_3_H and TpBD-(SO_3_H)_2_; Figure S5: TGA curves of TpPa-1, TpPa-SO_3_H and TpBD-(SO_3_H)_2_ under N_2_ atmosphere; Figure S6: Photographs demonstrating the hydrophilicity of TpPa-1, TpPa-SO_3_H and TpBD-(SO_3_H)_2_ with a water contact angle of 40°, 35°, 31°, respectively; Figure S7: SO_2_ sorption isotherms of (a) TpPa-1, (b) TpPa-SO_3_H and (c) TpBD-(SO_3_H)_2_ at 273 K and 298 K. Filled and open symbols indicate the adsorption and desorption, respectively; Figure S8: Virial analysis for SO_2_ adsorption isotherms of TpPa-1, TpPa-SO_3_H and TpBD-(SO_3_H)_2_ at 273 and 298 K with the fitting parameters (virial coefficients) *a*_i_ and *b*_j_. The virial coefficients ai have the unit [K·mol^−1^]; Figure S9: CO_2_ sorption isotherms of (a) TpPa-1, (b) TpPa-SO_3_H and (c) TpBD-(SO_3_H)_2_ at 273 and 298 K. Filled and open symbols indicate the adsorption and desorption isotherms, respectively; Figure S10: Virial analysis for CO_2_ adsorption isotherms of TpPa-1, TpPa-SO_3_H and TpBD-(SO_3_H)_2_ at 273 and 298 K with the fitting parameters (virial coefficients) *a*_i_ and *b*_j_. The virial coefficients ai have the unit [K·mol^−1^]; Figure S11: CH_4_ sorption isotherms of TpPa-1, TpPa-SO_3_H and TpBD-(SO_3_H)_2_ at 298 K. Filled and open symbols indicate the adsorption and desorption isotherms, respectively; Figure S12: N_2_ sorption isotherms of TpPa-1, TpPa-SO_3_H and TpBD-(SO_3_H)_2_ at 298 K. Filled and open symbols indicate the adsorption and desorption isotherms, respectively; Figure S13: IAST selectivity for CO_2_/N_2_ mixtures with varying CO_2_ molar fractions in the gas phase at 298 K and 1 bar; Figure S14: Setup for humid SO_2_ exposure experiments. Note: a, SO_2_ sensor and hygrometer; b, N_2_ flowmeter; c, natrium metabisulfite solution (Na_2_S_2_O_5_); d, sodium chloride solution; e, sample; f, NaOH aqueous solution; Figure S15: Comparison of PXRD patterns of (a) TpPa-1, (b) TpPa-SO_3_H and (c) TpBD-(SO_3_H)_2_ before and after exposure to dry or humid SO_2_ for different durations; Figure S16: Comparison of FT-IR patterns of (a) TpPa-1, (b) TpPa-SO_3_H and (c) TpBD-(SO_3_H)_2_ before and after exposure to dry or humid SO_2_ for different durations; Figure S17: Comparison of N_2_ adsorption isotherms of (a) TpPa-1, (b) TpPa-SO_3_H and (c) TpBD-(SO_3_H)_2_ before and after exposure to dry or humid SO_2_ for different durations; Figure S18: The retained BET surface area percentages of TpPa-1, TpPa-SO_3_H and TpBD-(SO_3_H)_2_ after exposure to dry or humid SO_2_ for different durations relative to corresponding pristine COFs; Table S1: CO_2_, CH_4_ and N_2_ uptakes at different partial pressures and isosteric enthalpies of adsorption on TpPa-1, TpPa-SO_3_H and TpBD-(SO_3_H)_2_ at 298 K; Table S2: Parameters of DSLAI Sips model fitted adsorption isotherms; Table S3: The result of IAST selectivity of TpPa-1, TpPa-SO_3_H and TpBD-(SO_3_H)_2_ at 298 K at 1 bar; Table S4: Comparison of SO_2_ sorption data in this work with reported COFs; Table S5: The breakthrough time of TpPa-1, TpBD-(SO_3_H)_2_ and representative adsorbents.
