# Supplementary material for: Isoreticular Synthesis of Ionic Covalent Organic Frameworks for Enhanced SO2 Adsorption and Separation
Source: Molecules. 2026 Apr 27;31(9):1445. doi: 10.3390/molecules31091445 (PMC13164858; doi:10.3390/molecules31091445)
Supplement: Supplementary file 1 [file molecules-31-01445-s001.zip › molecules-4250209-supplementary.pdf]

Supplementary Materials:

Isoreticular Synthesis of Ionic Covalent Organic Frameworks for Enhanced SO_2 Adsorption and Separation

Zhijie Liu[a], Shize Li[a], Jun Liang*[a,b], Qiao Wu*[a], Ruihu Wang*[a,b]

[a]Hebei Key Laboratory of Functional Polymer, School of Chemical Engineering and Technology, Hebei University of Technology, Tianjin 300401, China
[b]State Key Laboratory of Structural Chemistry, Fujian Institute of Research on the Structure of Matter, Chinese Academy of Sciences, Fuzhou, 350002, China

Section S1. Isosteric enthalpy of adsorption

Virial analysis of adsorption isotherms

From the comparison of calculating the isosteric enthalpy of adsorption ($-\Delta H_{ads}$) by different approaches, in line with our previous work the virial analysis was superior to the Freundlich–Langmuir fit/Clausius-Clapeyron approach to obtain rational values [1].

Hence, to calculate the isosteric enthalpy of adsorption from the gas isotherm data, the virial method was used. Equation (1) was used to fit the adsorption isotherms simultaneously at 273 and 298 K in the program Origin.

$$\ln(P) = \ln(n) + \frac{1}{T}\sum_{i=0}^{n} a_i n^i + \sum_{j=0}^{m} b_j m^j \qquad (1)$$

In equation (1), P is the pressure in kPa, n is the total amount adsorbed in mmol·g^{-1}, T is the temperature in K (here 273 K, 298 K), a_i and b_i are virial coefficients, and n and m represent the number of coefficients required to adequately fit the isotherms. Then, ΔH_{ads} can be calculated from equation (2), where R is the universal gas constant.

$$-\Delta H_{ads} = -R\sum_{i=0}^{n} a_i n^i \qquad (2)$$

Section S2. Ideal adsorbed solution theory (IAST) Selectivity

Selectivity of SO_2 over CO_2 of TpPa-1, TpPa-SO_3H and TpBD-$(SO_3H)_2$ were calculated from Dual Site Langmuir (DSL) (eq.3) fitted adsorption isotherm data.

$$q_{eq} = q_{max1}\frac{K_1 \cdot p}{1+K_1 \cdot p} + q_{max2}\frac{K_2 \cdot p}{1+K_2 \cdot p} \qquad (3)$$

The MixManager software (MixManager, China) calculates the maximal loadings of each gas depending on the given mole fraction.

IAST selectivities S of binary gas mixtures were calculated using equation 4, where x_i represents the absorbed gas amount and y_i the mole fraction of each adsorptive.

$$S = \frac{x_1/x_2}{y_1/y_2} \qquad (4)$$

Selectivities of SO_2 over CO_2, SO_2 over CH_4, SO_2 over N_2, CO_2 over CH_4, and CO_2 over N_2 of TpPa-1, TpPa-SO_3H and TpBD-$(SO_3H)_2$ were calculated from the DSL (eq. 3) fitted isotherm data. IAST with DSLangmuir isotherm mode was chosen, and the total pressure was fixed at 1 bar to give the IAST selectivity versus the SO_2 molar fractions between 0.02 and 0.5 bar in gas mixtures. Alternatively, SO_2 to CO_2 (or SO_2 to CH_4, SO_2 to N_2, CO_2 to CH_4, CO_2 to N_2) volume ratio was fixed to give the IAST selectivity vs pressure between 0.1 to 1.0 bar based on eq.4.

Section S3. Characterization and analysis

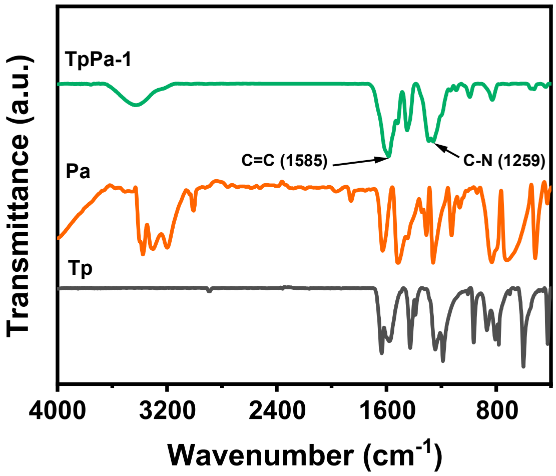

Figure S1. FT-IR spectra of Tp, Pa and TpPa-1.

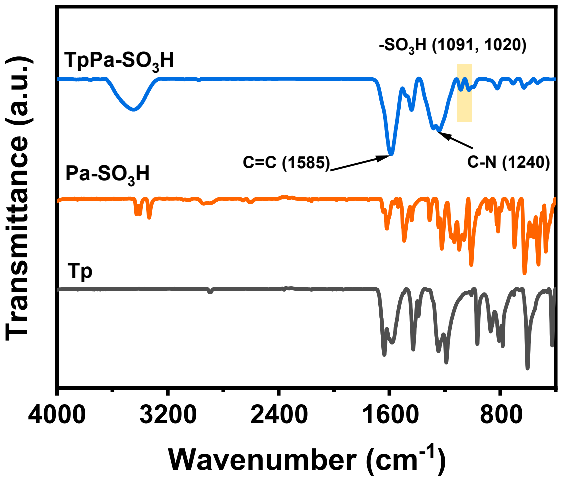

Figure S2. FT-IR spectra of Tp, Pa-SO3H and TpPa-SO3H.

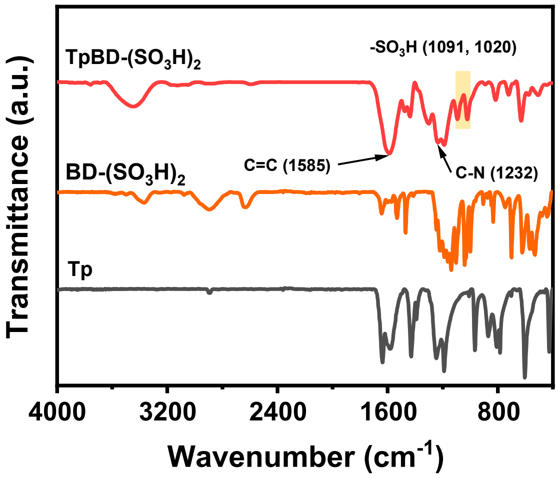

Figure S3. FT-IR spectra of Tp, BD-(SO_3H)$_2$ and TpBD-(SO_3H)$_2$.

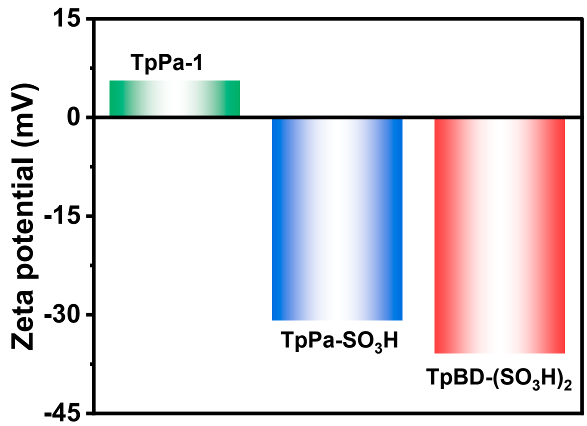

Figure S4. Zeta potential of TpPa-1, TpPa-SO_3H and TpBD-$(SO_3H)_2$.

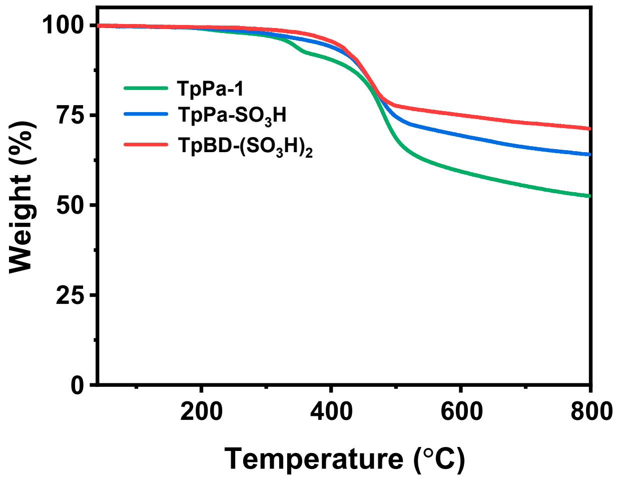

Figure S5. TGA curves of TpPa-1, TpPa-SO_3H and TpBD-$(SO_3H)_2$ under N_2 atmosphere.

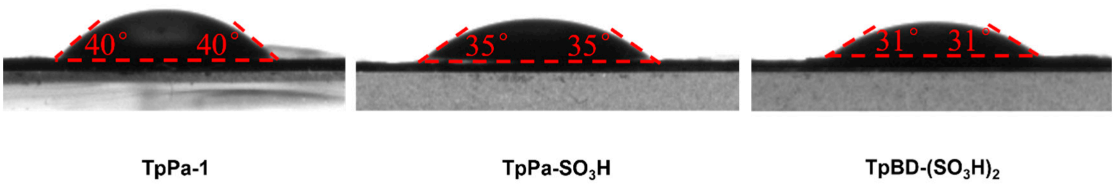

Figure S6. Photographs demonstrating the hydrophilicity of TpPa-1, TpPa-SO_3H and TpBD-$(SO_3H)_2$ with a water contact angle of 40°, 35°, 31°, respectively.

Section S4. SO_2, CO_2, CH_4 and N_2 gas adsorption experiments

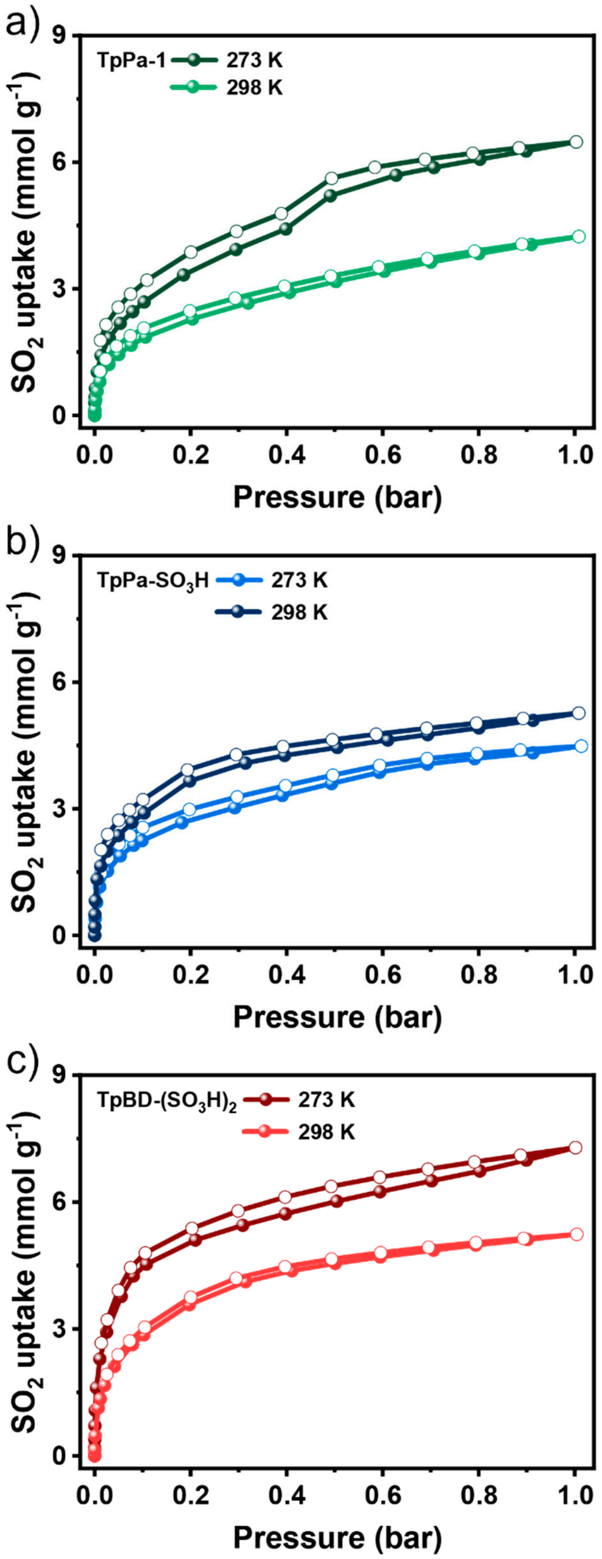

Figure S7. SO_2 sorption isotherms of a) TpPa-1, b) TpPa-SO_3H and c) TpBD-(SO_3H)$_2$ at 273 K and 298 K. Filled and open symbols indicate the adsorption and desorption, respectively.

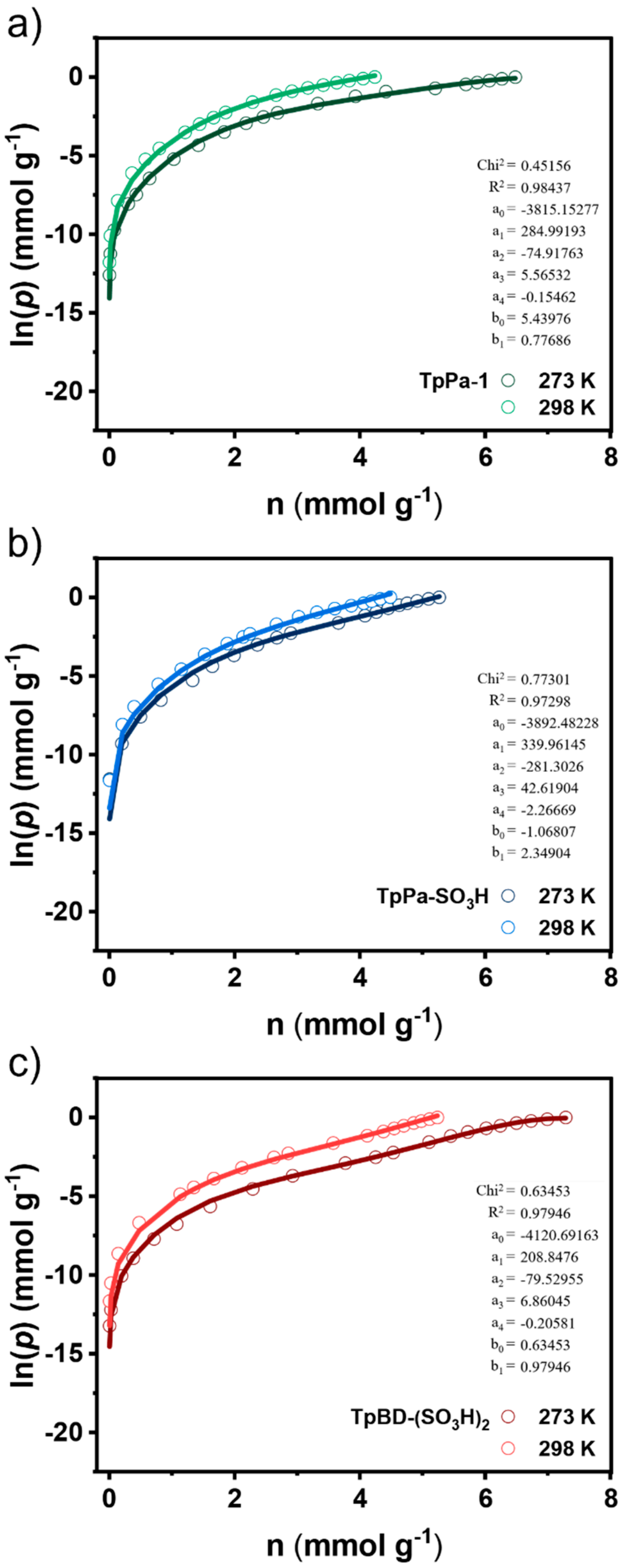

Figure S8. Virial analysis for SO_2 adsorption isotherms of TpPa-1, TpPa-SO_3H and TpBD-$(SO_3H)_2$ at 273 and 298 K with the fitting parameters (virial coefficients) a_i and b_j. The virial coefficients a_i have the unit [K·mol^{-1}].

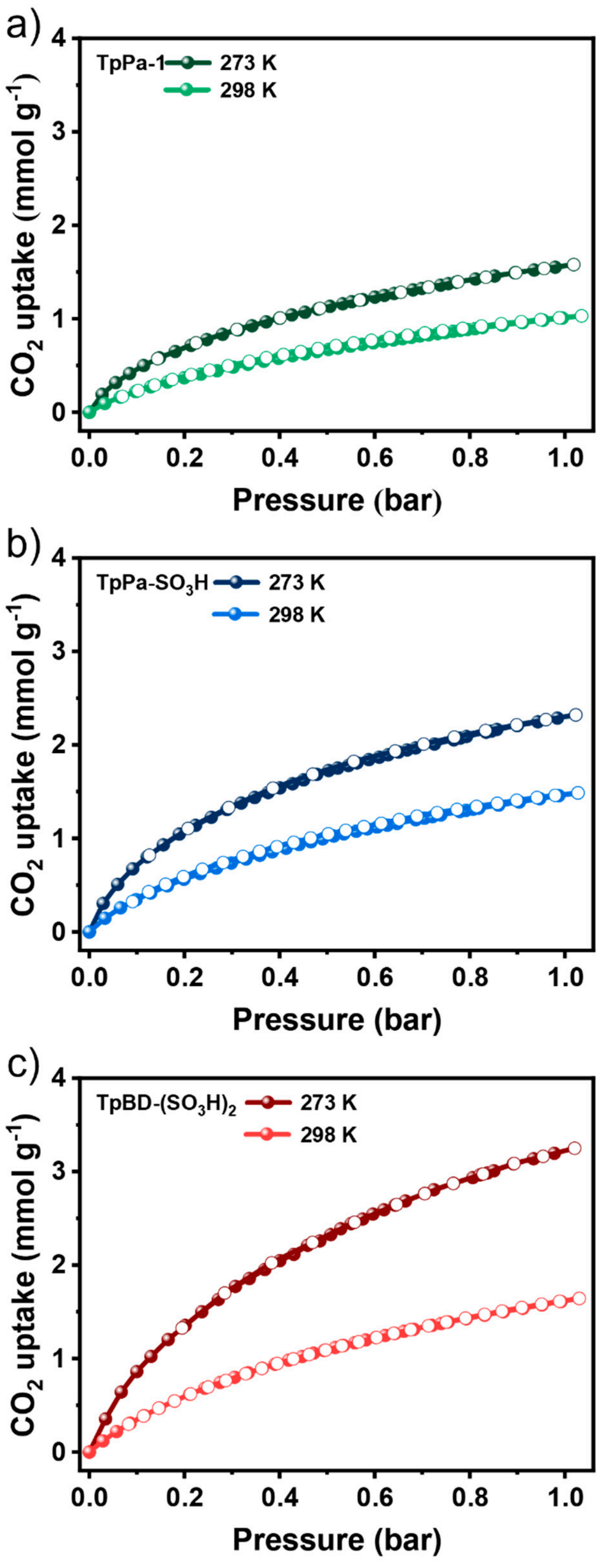

Figure S9. CO_2 sorption isotherms of a) TpPa-1, b) TpPa-SO_3H and c) TpBD-$(SO_3H)_2$ at 273 and 298 K. Filled and open symbols indicate the adsorption and desorption isotherms, respectively.

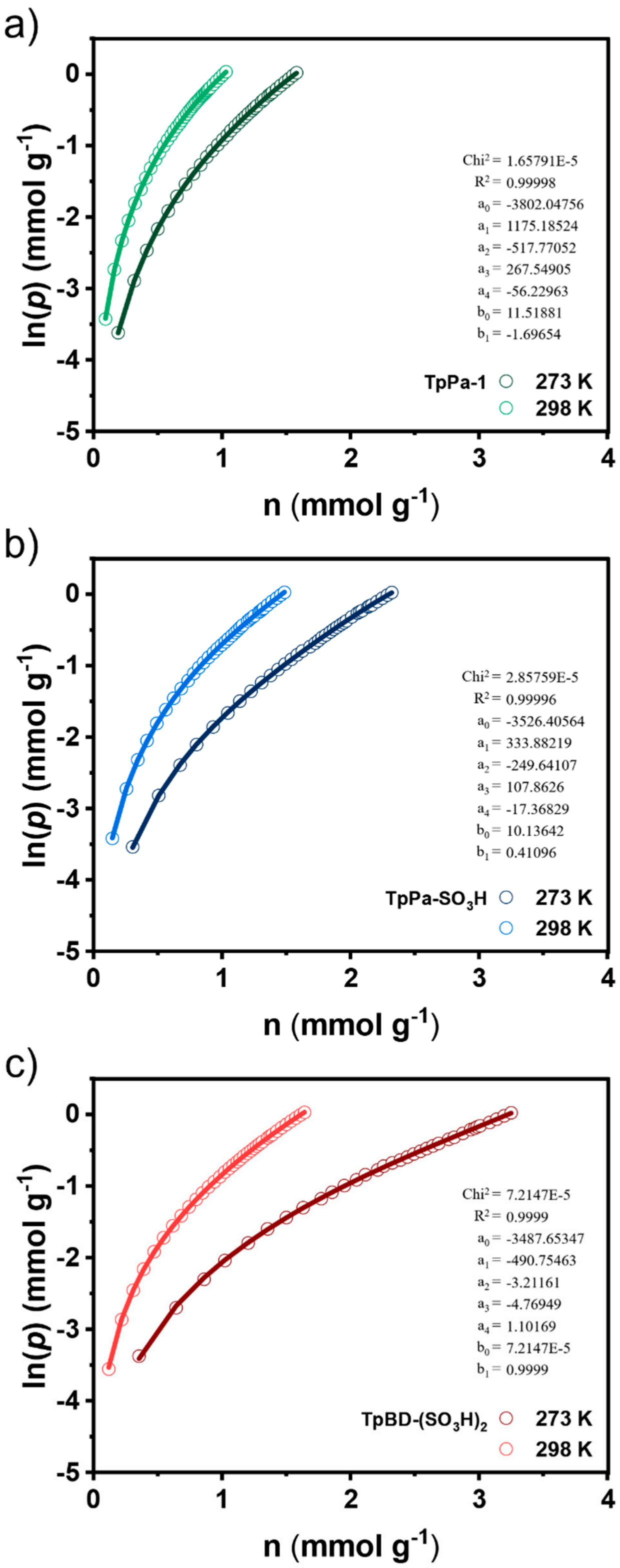

Figure S10. Virial analysis for CO_2 adsorption isotherms of TpPa-1, TpPa-SO_3H and TpBD-$(SO_3H)_2$ at 273 and 298 K with the fitting parameters (virial coefficients) a_i and b_j. The virial coefficients a_i have the unit [K·mol^{-1}].

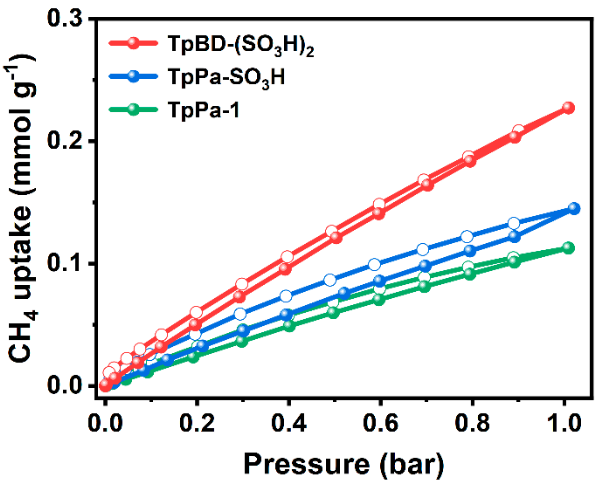

Figure S11. CH_4 sorption isotherms of TpPa-1, TpPa-SO_3H and TpBD-$(SO_3H)_2$ at 298 K. Filled and open symbols indicate the adsorption and desorption isotherms, respectively.

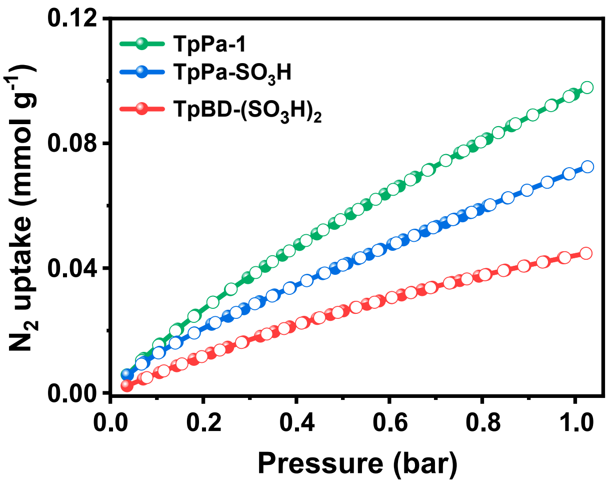

Figure S12. N_2 sorption isotherms of TpPa-1, TpPa-SO_3H and TpBD-$(SO_3H)_2$ at 298 K. Filled and open symbols indicate the adsorption and desorption isotherms, respectively.

Table S1. CO_2, CH_4 and N_2 uptakes at different partial pressures and isosteric enthalpies of adsorption on TpPa-1, TpPa-SO_3H and TpBD-$(SO_3H)_2$ at 298 K.

Materials	CO_2 uptake (mmol g^{-1}) 1.0 bar	CH_4 uptake (mmol g^{-1}) 1.0 bar	N_2 uptake (mmol g^{-1}) 1.0 bar
TpPa-1	1.03	0.11	0.096
TpPa-SO_3H	1.49	0.145	0.068
TpBD-$(SO_3H)_2$	1.64	0.22	0.042

Table S2. Parameters of DSLAI Sips model fitted adsorption isotherms.

COF	Gas	Tempe-rature [K]	R^2	Affinity constant K_1 [1/bar]	Maximal loading q_{max1} [mmol/g]	Affinity constant K_2 [1/bar]	Maximal loading q_{max2} [mmol/g]
TpPa-1	SO_2	298	0.9995	109.203	1.46189	0.739886	6.46013
	CO_2	298	0.9999	8.4512	0.265596	0.396684	2.72424
	CH_4	298	0.9999	0.15127	0.425775	0.15127	0.425767
	N_2	298	0.9964	0.073481	1.09217	3.31228	0.0276693
TpPa-SO_3H	SO_2	298	0.9989	222.335	1.64253	1.56322	4.61225
	CO_2	298	0.9996	9.88995	0.339861	0.663163	2.89705
	CH_4	298	0.9992	5.4964	0.007404	0.002591	51.4248
	N_2	298	0.9978	11.063	0.0117023	0.0633352	1.01159
TpBD-$(SO_3H)_2$	SO_2	298	0.9997	292.708	1.49508	4.4486	4.49546
	CO_2	298	0.9999	4.40744	0.611184	0.522645	3.25402
	CH_4	298	0.9999	38.6449	0.0021837	0.120153	2.08071
	N_2	298	0.9986	0.804247	0.00229	0.468788	0.134892

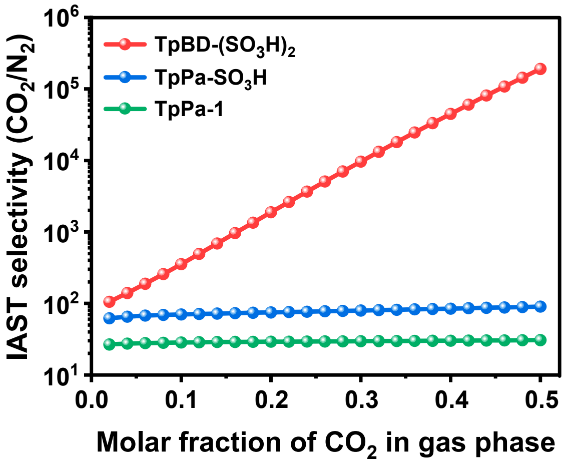

Figure S13. IAST selectivity for CO_2/N_2 mixtures with varying CO_2 molar fractions in the gas phase at 298 K and 1 bar.

Table S3. The result of IAST selectivity of TpPa-1, TpPa-SO_3H and TpBD-$(SO_3H)_2$ at 298 K at 1 bar.

Materials	IAST selectivity[a]				
	SO_2/CO_2 (10:90)	SO_2/CH_4 (10:90)	SO_2/N_2 (10:90)	CO_2/CH_4 (10:90)	CO_2/N_2 (10:90)
TpPa-1	51	157	182	24	28
TpPa-SO_3H	54	157	303	29	70
TpBD-$(SO_3H)_2$	61	124	621	17	355

[a] Constant pressure with varied molar ratio.

Table S4. Comparison of SO_2 sorption data in this work with reported COFs.

Materials	S_{BET} (m^2 g^{-1})	SO_2 uptake ($mmol\ g^{-1}$) [a]			Temp (K)	SO_2/CO_2 (molar ratio, x:y) [b]	Q_{st}	Ref.
		0.01 bar	0.1 bar	1.0 bar				
TpPa-1	686	0.78	1.82	4.24	298	51	31	This work
TpPa-SO_3H	195	1.19	2.25	4.46	298	54	32	This work
TpBD-$(SO_3H)_2$	429	1.27	2.83	5.24	298	61	34	This work
PI-COF-m	1003	-	-	6.50	298	-	-	[2]
CTF-CSU41	333			6.70	273	-	-	[3]
XJ-COF-2	961	-	-	11.07	298	83 (10:90)	-	[4]
Sono COF-9	1147	-	0.89	3.48	298	13 (10:90)	42	[5]
NKCOF-12	536	1.78	4.05	9.82	298	-	34	[6]
COF-701	1636	0.23	1.33	9.73	298	-	-	[6]
TMT-TA	1436	0.32	2.03	13.13	298	-	-	[6]
PZ-TPPT-COF	1023	-	-	9.60	298	36 (10:90)	-	[7]
PA-TFPT-COF	11	-	-	3.79	298	22 (10:90)	-	[7]
TzDa-COF	2100	-	-	12.28	298	19 (10:90)	32	[8]
TzBp-COF	346			2.84	298	14 (10:90)	42	[8]

[a] Data based on the first adsorption runs. [b] Data based on IAST calculations.

Section S5. Stability of crystallinity and porosity after dry and humid SO_2 exposure

Experimental details: For the dry exposure, a SO_2 isotherm was measured. For the humid SO_2 exposure experiment, we used a similar setup (**Figure S14**) to Walton et al [9]. A controlled N_2 flow of 2 L min^{-1} was bubbled through a sodium hydrogen sulfite solution (0.4 g $Na_2S_2O_5$ in 100 mL water) in a round bottom flask to transport gaseous SO_2 into a humidity chamber (a desiccator vessel). The desiccator was equipped with a crystallizing dish filled with saturated sodium chloride solution (80 mL, relative humidity (RH) 75%) and an open vial filled with the sample (50 mg). The RH and the amount of SO_2 in the desiccator was monitored with a hygrometer and SO_2-sensor (KEERUNO GT903-SO_2-B Portable sulfur dioxide detector), synchronously.

TpPa-1, TpPa-SO_3H and TpBD-$(SO_3H)_2$ were exposed to a humid SO_2 environment at room temperature with 75 ± 6% RH and 35 ± 5 ppm SO_2 for 6 h. The crystallinity (PXRD), FT-IR and BET surface area before and after dry and humid SO_2 exposure were measured, and the results are given below in **Figure S15-S17**.

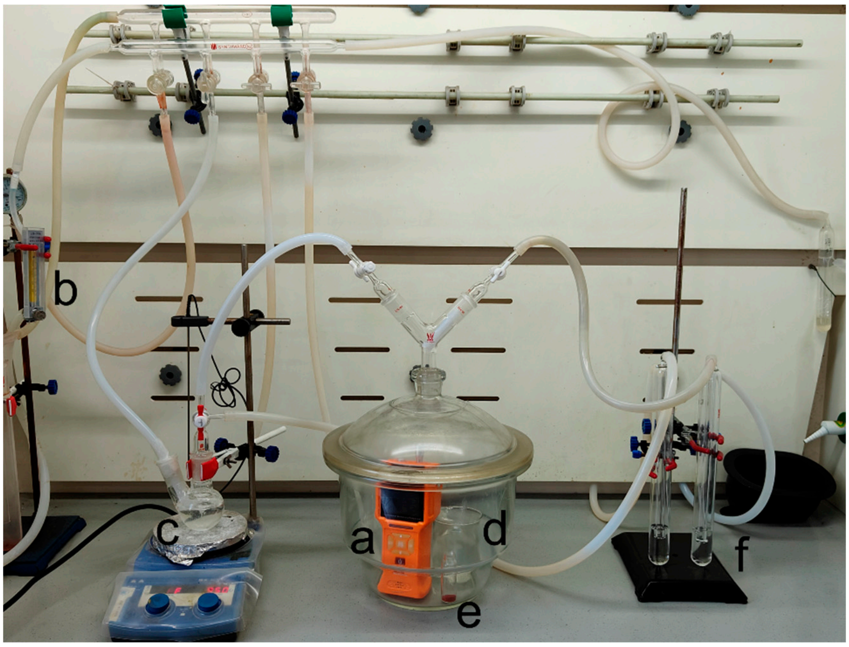

Figure S14. Setup for humid SO_2 exposure experiments. Note: a, SO_2 sensor and hygrometer; b, N_2 flowmeter; c, natrium metabisulfite solution ($Na_2S_2O_5$); d, sodium chloride solution; e, sample; f, NaOH aqueous solution.

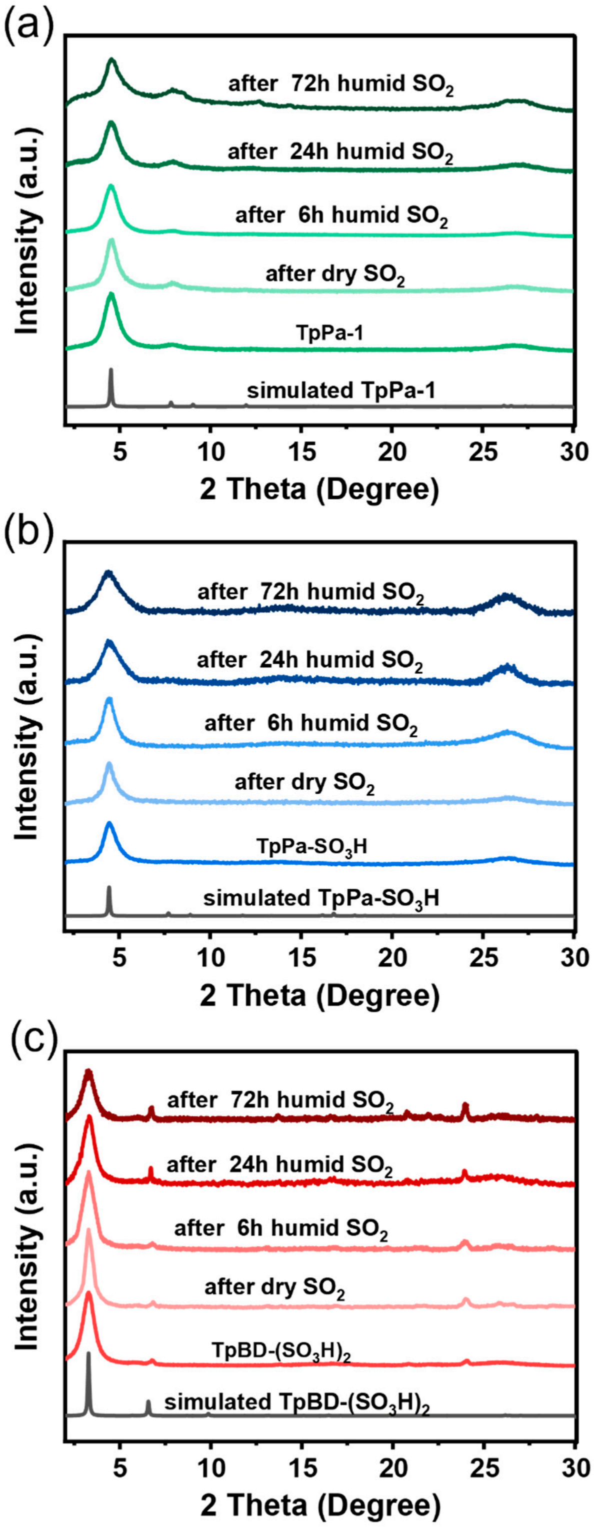

Figure S15. Comparison of PXRD patterns of a) TpPa-1, b) TpPa-SO_3H and c) TpBD-$(SO_3H)_2$ before and after exposure to dry or humid SO_2 for different durations.

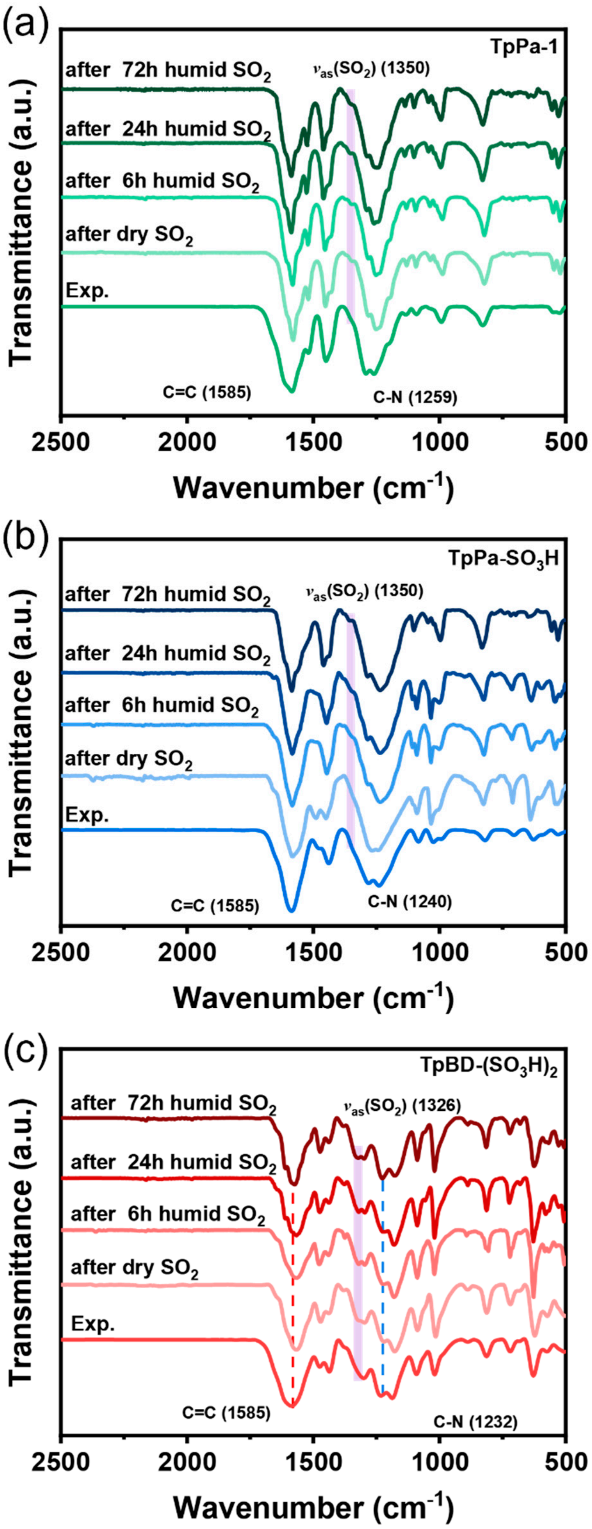

Figure S16. Comparison of FT-IR patterns of a) TpPa-1, b) TpPa-SO_3H and c) TpBD-$(SO_3H)_2$ before and after exposure to dry or humid SO_2 for different durations.

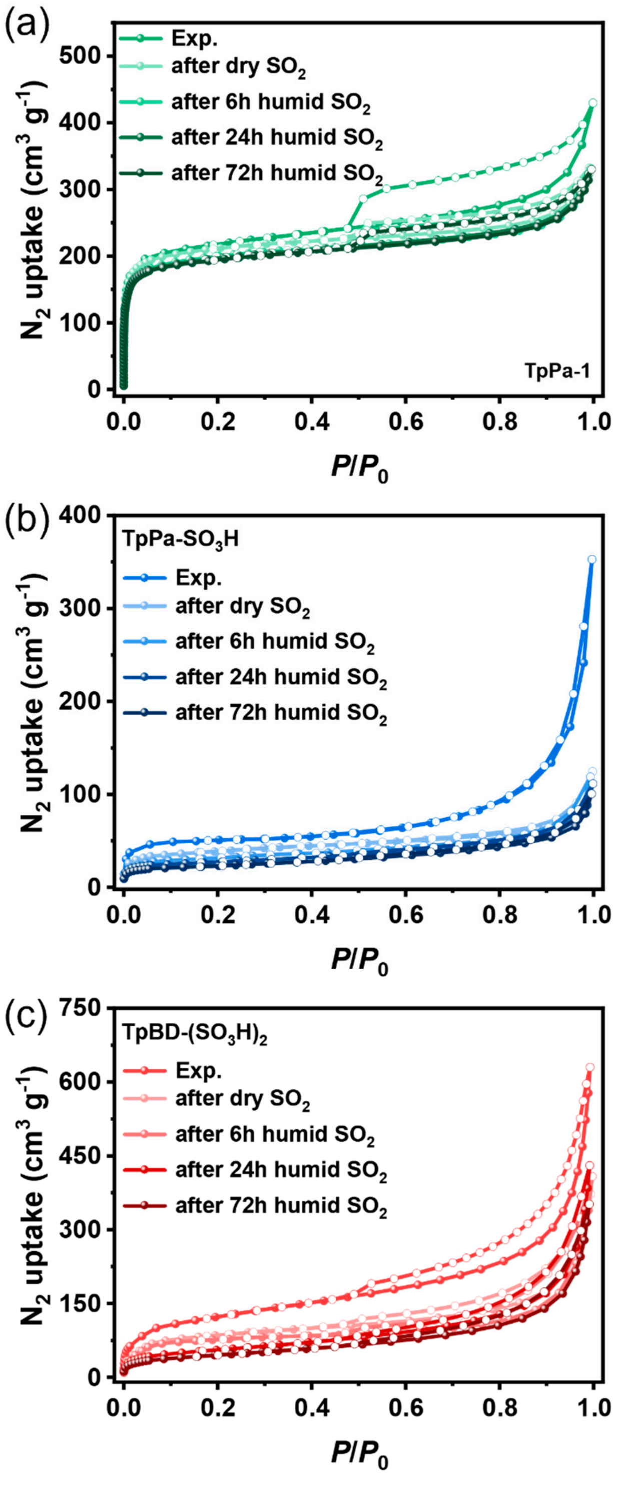

Figure S17. Comparison of N_2 adsorption isotherms of a) TpPa-1, b) TpPa-SO_3H and c) TpBD-$(SO_3H)_2$ before and after exposure to dry or humid SO_2 for different durations.

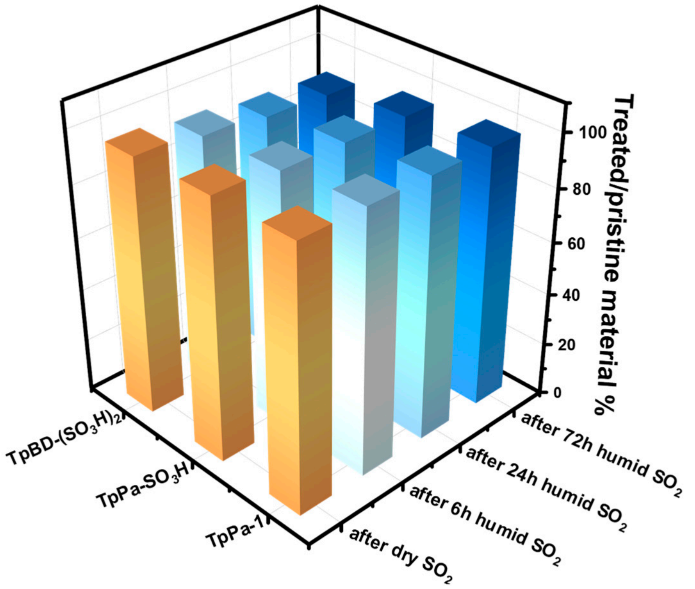

Figure S18. The retained BET surface area percentages of TpPa-1, TpPa-SO_3H and TpBD-$(SO_3H)_2$ after exposure to dry or humid SO_2 for different durations relative to corresponding pristine COFs.

Section S6. Breakthrough performance

Table S5. The breakthrough time of TpPa-1, TpBD-$(SO_3H)_2$ and representative adsorbents.

Material	Composition of gas mixture SO_2 (ppm)	CO_2 (v:v %)	N_2 (v:v %)	Inlet flow rate (mL min^{-1})	Breakthrough time (min g^{-1})	Ref.
TpPa-1	2000	14.8	85	8	311	This work
TpBD-$(SO_3H)_2$	2000	14.8	85	8	987	This work
NKCOF-12	2000	14.8	85	6	911	[6]
COF-701	2000	14.8	85	6	43	[6]
TMT-TA	2000	14.8	85	6	66	[6]
Activated carbon fiber felt	2000	14.8	85	6	392	[6]
ZSM-5	2000	14.8	85	6	166	[6]
Diatomite	2000	14.8	85	6	35	[6]
PZ-TPPT-COF	2000	15	84.8	10	100	[7]
TzDa-COF	500	15	84.8	10	200	[8]

Section S7. References

1. Nuhnen, A.; Janiak, C. A practical guide to calculate the isosteric heat/enthalpy of adsorption via adsorption isotherms in metal-organic frameworks, MOFs. *Dalton Trans.* **2020,** *49*, 10295–10307.
2. Lee, G.; Lee, J.; Vo, H.; Kim, S.; Lee, H.; Park, T. Amine-functionalized covalent organic framework for efficient SO_2 capture with high reversibility. *Sci. Rep.* **2017,** *7*, 557.
3. Fu, Y.; Wang, Z.; Li, S.; He, X.; Pan, C.; Yan, J.; Yu, G. Functionalized covalent triazine frameworks for effective CO_2 and SO_2 removal. *ACS Appl. Mater. Interfaces* **2018,** *10*, 36002–36009.
4. Fu, Y.; Wu, Y.; Chen, S.; Zhang, W.; Zhang, Y.; Yan, T.; Yang, B.; Ma, H. Zwitterionic covalent organic frameworks: attractive porous host for gas separation and anhydrous proton conduction. *ACS Nano* **2021,** *15*, 19743–19755.
5. Zhao, W.; Obeso, J.; López-Cervantes, V.; Bahri, M.; Sánchez-González, E.; Amador-Sánchez, Y.; Ren, J.; Browning, N.; Peralta, R.; Barcaro, G.; Monti, S.; Solis-Ibarra, D.; Ibarra, I. A.; Zhao, D. Achieving sub-ppm sensitivity in SO_2 detection with a chemically stable covalent organic framework. *Angew. Chem. Int. Ed.* **2025,** *64*, e202415088.
6. Wang, S.; Fu, Y.; Wang, F.; Wang, X.; Yang, Y.; Wang, M.; Wang, J.; Lin, E.; Ma, H.; Chen, Y.; Cheng, P.; Zhang, Z. Scalable melt polymerization synthesis of covalent organic framework films for room temperature low-concentration SO_2 detection. *J. Am. Chem. Soc.* **2024,** *146*, 33509–33517.
7. Qu, Y.; Liu, F.; Zhuo, L.; Zheng, L.; Zhong, S.; Xiao, Y.; Zheng, Y.; Zheng, A.; Liu, F.; Jiang, L. Pyrazine-functionalized sp^2 carbon-conjugated covalent organic frameworks for selective capture of sulfur dioxide. *Sep. Purif. Technol.* **2025,** *371*, 133402.
8. Zhuo, L.; Qu, Y.; Zhong, S.; Zheng, L.; Xiao, Y.; Zheng, Y.; Liu, F.; Jiang, L. Covalent organic framework for room-temperature pressure swing adsorption of SO_2. *Chem. Eng. Sci.* **2026,** *320*, 122593.
9. Mounfield, W., Iii; Han, C.; Pang, S.; Tumuluri, U.; Jiao, Y.; Bhattacharyya, S.; Dutzer, M.; Nair, S.; Wu, Z.; Lively, R.; Sholl, D.; Walton, K. Synergistic effects of water and SO_2 on degradation of MIL-125 in the presence of acid gases. *J. Phys. Chem. C* **2016,** *120*, 27230–27240.
